# The development of a Community Service Announcement to raise awareness of the Home Medicines Review health service program

**DOI:** 10.1111/ajag.70086

**Published:** 2025-08-28

**Authors:** Hui Wen Quek, Deborah Hawthorne, Esther Hernandez, Angus Thompson, Georgie B. Lee, Bente Hart, Karalyn Huxtagen, Prasin Rodrigues, Anna Barwick, Tiernan McDonough, Faith Young, Brett Curie, Katie Phillips, Raymond Truong, Diana Ly, Manya Angley, Amy T. Page

**Affiliations:** ^1^ Centre for Optimisation of Medicines, School of Health and Clinical Sciences The University of Western Australia Crawley Western Australia Australia; ^2^ Consultant Clinical Pharmacist New South Wales Queensland Australia; ^3^ School of Health The University of New England Armidale New South Wales Australia; ^4^ Clinical and Health Sciences University of South Australia Adelaide South Australia Australia; ^5^ School of Pharmacy The University of Queensland Woolloongabba Queensland Australia; ^6^ Manya Angley Research and Consulting South Australia Australia

**Keywords:** Evidence‐Based Pharmacy Practice, Health Services Research, Medication Review, Medication Therapy Management, Pharmacists

## Abstract

Home Medicines Reviews (HMRs) conducted by credentialed pharmacists in response to referrals from medical practitioners are funded by the Australian government to improve the quality use of medicines. In late 2023, a grassroots group of credentialed pharmacists created a Community Service Announcement (CSA) to raise consumer awareness of the HMR program. Community Service Announcements are allocated airtime by mainstream media (e.g. television and radio stations) for promoting messaging in the public interest, such as health services. The HMR awareness CSA was funded by 250 donors through a GoFundMe campaign. Two days of filming with pharmacists, doctors and patients across three rural and regional areas resulted in a 28‐s video and corresponding radio sound bites. The CSA aired on major national Australian broadcast networks with coverage during both on‐peak and off‐peak times across all states and territories. The total value of the television advertising was estimated at AUD1.65 million. Radio messages were aired 36 times over a week on stations nationwide, reaching an estimated 1,911,300 listeners, approximately 8% of the Australian population. This CSA campaign illustrated the potential of health professional initiatives to raise awareness of government health programs. By leveraging crowdfunding and community support, this activity demonstrated a model for other health professionals seeking to promote similar health promotion and awareness initiatives.

**Practice impact**

This Community Service Announcement (CSA) Home Medicines Review (HMR) awareness campaign demonstrates the potential of grassroots initiatives to enhance awareness of health services.

**Policy impact**

A Community Service Announcement (CSA) disseminates a message that the broadcaster provides free of charge to support the community. This HMR CSA campaign illustrates the potential of health professional initiatives to raise awareness of government programs.

## 1 INTRODUCTION

Medication safety is a priority not just for consumers, but for health‐care providers and policymakers alike. In Australia, there are estimated to be 250,000 hospital admissions and 400,000 emergency department presentations each year that are medication‐related.^1^ The associated cost is estimated to be AUD1.4 billion, and 50% are potentially preventable.^1^ The Home Medicines Review (HMR) program is intended to assist consumers and their health‐care teams to optimise medication regimens, supporting current health needs and preventing possible future medication‐related harm and errors.^2^

Home Medicines Reviews are an Australian Government‐funded collaborative health service provided by credentialed pharmacists on referral from a medical practitioner (most commonly a general practitioner).^2^ The HMR program has existed for over 20 years, but throughout this time, the provision of HMRs by credentialed pharmacists has faced many challenges, including but not limited to monthly service caps on the number of reviews each credentialed pharmacist can perform, a recent lack of indexation of payments to credentialed pharmacists performing HMRs, termination of the COVID response telehealth delivery of HMRs and closure of the original accrediting body, the Australian Association of Consultant Pharmacy (AACP).^3^ There are also known barriers to medical practitioners referring patients for HMRs. Amongst these are sub‐optimal awareness of the service, time constraints and the administrative burden.^4^ Government data shows that in 2023, general practitioners (GPs) claimed for only 90,000 HMRs through Medicare Benefits Schedule (Item 900) billings, which funds the referral and review components of a HMR,^5^ while credentialed pharmacists who performed the HMRs claimed for 130,000 HMRs under a separate funding stream, the Pharmacy Programs Administrator (PPA).^6^ It is unclear what is behind this discrepancy, although it suggests reasonable attrition through the phases of HMRs with the need for multiple appointments. These issues may impede the broad uptake of HMR program delivery, resulting in underserviced areas in Australia and a missed opportunity to advance the medication safety agenda. A previous study suggested that direct‐to‐consumer promotion of HMRs would increase the uptake of this service.^7^

A Community Service Announcement (CSA) is an evidence‐based public health promotion strategy that leverages mass media to raise awareness, increase knowledge and influence attitudes. Mass media campaigns are widely recognised as a cost‐effective way to deliver targeted messages to large audiences through established channels such as television, radio and newspapers. These campaigns have been successfully used to address a range of public health matters, including smoking cessation, road safety and cancer screening.^8^ Research shows that such campaigns can produce measurable improvements in health‐related behaviours and help prevent adverse outcomes.^8^ The Canadian Deprescribing Network has previously employed a similar media coverage strategy to disseminate messages as part of a multipronged approach to increase public awareness, engagement and action around deprescribing. This strategy has led to increased website traffic and broader public interest in the topic.^9^

Behaviour change is a complex process that often requires a multifaceted approach, including environmental or policy changes.^10^ Mass media campaigns such as CSAs help establish a foundation for subsequent behaviour change.^10^, ^11^ Community Service Announcements are underpinned by behaviour change theories which help conceptualise how individuals may progress towards desired actions.^12^ One such framework is the Transtheoretical Model, which outlines behavioural change as a series of stages: precontemplation, contemplation, preparation, action, maintenance and termination.^13^ Evidence from a past smoking cessation campaign suggests that individuals in the preaction stage are particularly responsive to media messages focused on increasing awareness and knowledge.^14^ This supports the strategic value of CSAs for initiating early steps in the behaviour change process.

This paper describes the development of a CSA initiated by a grassroots group of credentialed pharmacists, aimed at raising community awareness of the HMR program and encouraging consumers to discuss the service with their GP if they believed it could benefit them.

## 2 INNOVATION

### 2.1 Community Service Announcements (CSAs)

In Australian media, CSAs are short broadcasts designed to inform and educate the public about important social, health or safety issues.^15^ Media organisations in Australia are encouraged, and sometimes obligated, to allocate a portion of their airtime to CSAs. This is part of their social responsibility and commitment to contributing to the public good and is typically aired free of charge by television and radio stations as part of their commitment to serving the community. The primary objective of CSAs was to raise awareness and promote positive behaviours or initiatives that are expected to benefit the public. By leveraging the wide reach of media outlets, CSAs can effectively disseminate critical messages to diverse and extensive audiences, thereby supporting various public health and social campaigns. This aligns with the aim to raise public awareness of the value and availability of HMR health service.

To develop a CSA, groups such as not‐for‐profit organisations, charities or community groups need to produce a high‐quality video and/or audio segment. After producing the CSA, the group submits it to media outlets. The media outlet assesses whether the CSA is a message that it will broadcast and then elects to schedule it for broadcast. This collaborative effort between community organisations and media providers intends to facilitate important messages to reach a broad audience, enhancing public awareness and fostering community well‐being.

### 2.2 Identified problem

An online community of practice, known as *Consultant Pharmacists Australia*, was established on 23 April 2020 and is hosted on Facebook. The goals of the group are to (1) provide a sense of community and (2) provide a platform for connection among credentialed pharmacists, most of whom work independently and may otherwise have limited access to peer support and professional interaction. Since its inception, the group now has over 3700 members and has become an active forum for credentialed pharmacists to discuss their anecdotal experiences in conducting medication reviews, business or clinical‐related problems, and share workload.

A recurring theme in group discussions is the low level of consumer awareness about HMR service prior to referral by a GP.^7^, ^16^ These credentialed pharmacists frequently report anecdotes that this lack of awareness contributes to initial reluctance from consumers to engage with the service. However, anecdotally, post‐HMR feedback often reveals that consumers found the service valuable and believe it should be more widely promoted. These real‐life experiences highlight public unfamiliarity with the HMR service as a key barrier to uptake. Given access to the service is currently dependent on GP initiation, its reach may be further limited.

### 2.3 Initiation

The shared concern within the *Consultant Pharmacists Australia* online community of practice sparked the idea for the CSA as an awareness initiative. From the outset, pharmacist members of the online community of practice were engaged. The members were introduced to the idea in an initial online post with an attached video in October 2023 containing a link to a GoFundMe page (Box 1). The members were updated through 10 separate posts, all of which had high engagement levels. A virtual meeting was held during the process from conception to launch of the CSA in the media. A Sydney‐based media production company quoted the cost price of filming and production for both the television advertisements and the radio announcements supporting the initiative. A GoFundMe campaign was set up to raise the required funds (Box 1). Within 5 days, the campaign successfully raised the required amount from 250 unique donors. Two donors left words of support, stating ‘The Australian public need HMRs now more than ever,’ and ‘I am sick of hearing “I've never heard of this before! How long has this been around?” from healthcare professionals and patients alike’. As some contributions were anonymous, we were unable to confirm with certainty whether all contributions were from credentialed pharmacists.

BOX 1 **The GoFundMe campaign.**

**Table:** 

Did you know that a quarter of a million people are admitted to hospital every year due to medication misadventures? And a further 400,000 people visit the emergency department due a to medication‐related issues? What's even more important, is that up to 50% of these issues could be avoided. Hi, My name is Debbie and I am a pharmacist who lives in rural Victoria. I am a consultant pharmacist which means that I have completed post graduate training to be able to visit people in the comfort of their own homes to provide a Home Medication Review (HMR). HMRs are a government‐funded program to support the quality use of medicines—with **ZERO** out of pocket cost to the consumer. This means that a pharmacist will sit with a person in their home (along with their loved ones or carers beside them) to help them get the best out of their medications. This could be via education, device technique help, avoiding side effects or drug interactions, helping reduce the number of potentially inappropriate medications and so much more, all while communicating this back to the treating care team, and ensuring the patient and their wishes are at the centre. There is no age limit to being able to access this program, and there is no minimum number of medications required. However, as we know, Australia is an ageing population. People are living longer and with more chronic diseases than ever before. A HMR is one tool we can use to help people stay safe in their homes by helping them manage their medications to the best of their ability through a comprehensive medication review. HOWEVER—there is a big barrier to this program—**the majority of consumers don't know the essential HMR program even exists**! A referral to a pharmacist is made via their regular General Practitioner in most instances, and there is no advertising. We want to change this—we want to get the word out there to help inform consumers that this program exists. And they can ask their doctor to help them access it. Patients, carers and health‐care providers alike highly regard this program but it isn't utilised. Help us raise funds to be able to provide a consumer targeted campaign to allow people to get the most out of their medications through a Home Medication Review today! To find out more about the HMR program visit—https://www.ppaonline.com.au/programs/medication‐management‐programs/home‐medicines‐review
Link: https://www.gofundme.com/f/create‐a‐consumer‐home‐medication‐review‐campaign

### 2.4 Development

#### 2.4.1 Video

The video production involved 2 days of filming with a three‐member video production team, including the producer, editor and videographer. The on‐screen talents featured in the video included practising credentialed pharmacists, patients and GPs across three rural and regional areas: Benalla, Wangaratta and Wollongong. The result was a professionally produced 28‐s video. The filming process was shared with the *Consultant Pharmacists Australia* members on both days 1 and 2.

Still images were captured from the filming to highlight key messages for dissemination. The process of distilling the key messages was used as another opportunity to engage the members of *Consultant Pharmacists Australia* (Box 1).

#### 2.4.2 Radio

Corresponding radio sound bites (known as ‘grabs’) were prerecorded with individual messages with a contracted journalist from the media collective. The seven messages were recorded via phone with one credentialed pharmacist. The seven key messages that were recorded were:

1. ‘A Home Medicines Review service is a wonderful government funded, free service available to most Australians which involves the person taking the medication, family, carer, the regular doctor and specially trained pharmacist checking to ensure that all medications are working well for person taking them’.
2. ‘The GP will generate a referral which gets sent out to a specially trained pharmacist. The pharmacist will contact the person to visit them in their own home, conduct the review. Any issues that come out of that review, they'll send a report back to the GP. And then together with the pharmacist, the patient, and the GP will come up with a medication management plan going forward’.
3. ‘About 150,000 people in Australia each year are already accessing this service, which might sound like a lot but really there's probably at least a million people in Australia who take five or more medications regularly. And the evidence suggests that the more medications a person takes, unfortunately the higher the risk of adverse events and medication errors’.
4. ‘A quarter of a million people are admitted to hospital each year, on top of that almost half a million presentations to the emergency department are also thought to be due to medication problems – which is huge. And that's really what medication reviews are about, preventing potential harm from medications that you're taking now and into the future’.
5. ‘If you've ever felt unsure about the medications that you or a loved one is taking, or just want to feel more informed and confident about the medicines that you're putting into your body every day – we really encourage you to speak to your GP about getting a medication review done in your home’.
6. ‘There's a common misconception out there that medication reviews are only for older Australians, but a home medicines review is really relevant to anyone who takes regular medication regardless of age, regardless of where you live. And having that set of eyes by a specially trained medication review pharmacist, we believe, can only end in good results’.
7. ‘We really want to encourage local patients, as well as family and carers, to speak to their usual GP about accessing a Home Medicines Review. So, we've got specially trained medication review pharmacists all over Australia, in every area you can think of, who are ready and waiting to be able to visit you at home to ensure that the medicines you're taking are not only safe and appropriate but they're also looking forward to helping you get the best out of your medications’.

#### 2.4.3 Endorsement

**FIGURE 1:**
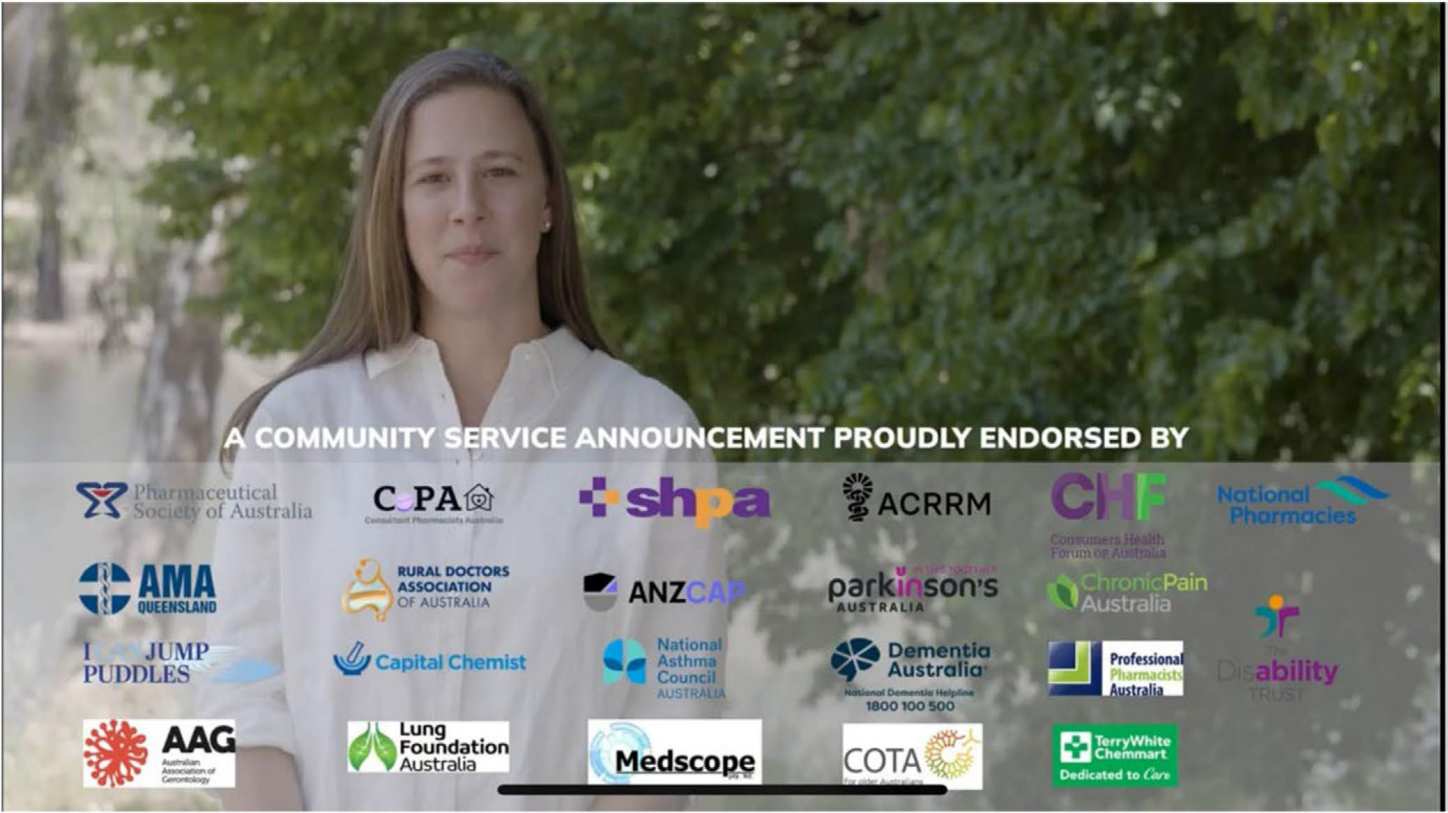
Endorsement of the Community Service Announcement.

The members of *Consultant Pharmacists Australia* group were again given the opportunity to engage with the process to seek endorsements for the CSA (Box 1). Of the organisations invited to endorse the CSA, 22 organisations agreed to use their logo endorsing the version that aired (Figure 1). The endorsing organisations included professional organisations (e.g. Pharmaceutical Society of Australia [PSA], Society of Hospital Pharmacists [SHPA], Australia Australian Association of Gerontology [AAG], Rural Doctors Association of Australia (RDA), Australian College of Rural and Remote Medicine [ACRRM] and the Australian Medical Association Queensland [AMA‐Q]), consumer organisations (Consumer Health Forum of Australia [CHF], Council of the Ageing [COTA]), pharmacy groups (e.g. Capital Chemist, National Pharmacies and Terry White Chemmart) and disease state organisations (e.g. Lung Foundation Australia, Parkinson's Australia, Dementia Australia, Disability Trust and Chronic Pain Australia).

### 2.5 Broadcast

The CSA was strategically aired on major television networks (Table 1) in metropolitan and regional areas. Coverage was secured during both on‐peak and off‐peak times across all states and territories, ensuring wide‐reaching exposure. The television advertising was valued at an estimated AUD530,000 for regional television stations and AUD1.12 million for metropolitan television stations, meaning that the value was an estimated AUD1.65 million. While no estimates were provided on the potential number of viewers reached, it was played in every capital city and regional area of every state and territory.

**TABLE 1:** Television airtime.

Television station	Off‐peak airtime %	Peak airtime %	Pattern	The value of media given at rate card, AUD
Metro national				
Channel 7	90	10	It was played in every state—with Perth and Brisbane having the highest frequency	480,000
Channel 10	100	0	Played in every state and was prominent in NSW	290,000
Channel 9	100	0	Not provided	350,000
Total				1,120,000
Regional national				
WIN	60	40	Played in every state except NT and was weighted in spots more to NSW and QLD	120,000
SCB	50	50	Played in every state and was weighted in spots more to VIC and SA	250,000
PRIME	80	20	Played in every state and was weighted in spots more to WA and NSW	160,000
Total				530,000

Abbreviations: NSW, New South Wales; NT, Northern Territory; QLD, Queensland; SA, South Australia; VIC, Victoria; WA, Western Australia.

Radio stations across Australia broadcast the CSA message. The radio messages aired 36 times over a week. The radio messages alone were estimated to reach up to 1,911,300 listeners, or up to 8% of all Australians.

The video as aired can also be viewed on YouTube at https://www.youtube.com/watch?v=c3VssXGcw6U. At the time of writing, the video has received over 1900 views and 33 likes. Viewer engagement included a positive public comment stating: ‘GREAT!! More people should be aware of the Medication Review service provided by [Credentialed] Pharmacists to ensure the quality use of medicines in community’. This suggests that the CSA is resonating with its intended audience and may be contributing to increased awareness of the service, in line with the campaign's goals.

### 2.6 Future directions

While the primary aim of the CSA was to raise awareness of HMR services, future research could explore whether the campaign contributes to increased awareness and subsequent uptake of HMR referrals. Measuring changes in referral rates over time may provide insight into behavioural change beyond the preaction stage. As a future direction, baseline data on HMR service use have been included to support future evaluation efforts. The crude rate of HMR referrals and GP billing for MBS item 900 were calculated quarterly between Q1 2015 and Q2 2024 using the Estimated Residential Population as the denominator. Trends were evaluated using joinpoint regression on the logarithmic scale, which estimated the Quarterly Percentage Change (QPC) with up to seven joinpoints allowable.

**FIGURE 2:**
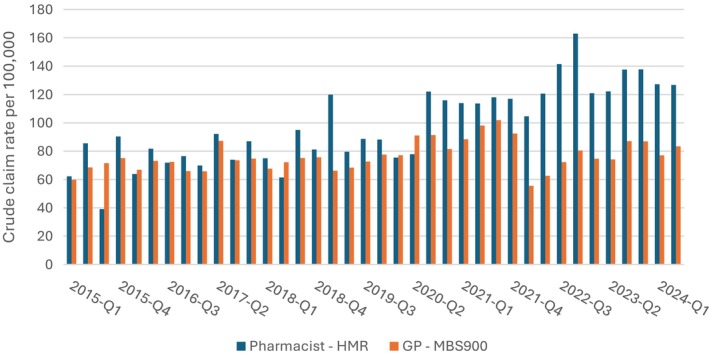
Crude quarterly rates of pharmacist Home Medicines Review (HMR) claims and general practitioner Medicare Benefits Schedule (MBS) item 900 claims per 100,000 population, Q1 2015 to Q1 2024.

A stable increase in the rate of HMR referrals was observed over the study period (+9% per quarter [95% CI: 6.5, 11.0]). While the rate of MBS item 900 claims fluctuated, with growth between Q2 2019 and Q2 2021 (+16% per quarter [95% CI: 4.0, 51.7]), a decline between Q2 2021 and Q1 2022 (−33% per quarter [95% CI: −47.9, −8.8]) and growth again from Q2 2022 onwards (+12% per quarter [95% CI: 1.0, 29.7]). When plotted side‐by‐side, the rate of GP billing for post‐HMR follow‐up appears to be outstripped by the consistent growth in HMR referrals, with the difference in crude rates increasing from late 2020 and more notably from 2022 onwards (Figure 2).

## 3 DISCUSSION

The CSA campaign likely fulfilled its primary objective to raise public awareness of the HMR program, as shown by the high level of media coverage and the substantial value of the in‐kind advertising, which contributed to a widespread dissemination of the message.

The success of the development of the CSA campaign underscores the potential of grassroots initiatives led by health professionals to enhance awareness and support for government programs. This may be attributed to the community appeal of government‐funded initiatives that have limited awareness among consumers. The campaign was made possible through the support of practising health professionals and their patients, along with the engagement of media collaborators and media outlets. The use of crowdfunding to finance the campaign demonstrated the feasibility of mobilising community resources to support public health initiatives.

The CSA initiative highlights the opportunity for grassroots practitioners to undertake crowdfunded activities to promote health and well‐being in the community. The success of the development of this campaign suggests that similar approaches could be employed to support other public health programs, thereby enhancing their reach and impact. The campaign highlighted the role of HMRs to consumers, explaining the program's role in promoting medication safety and optimising therapeutic outcomes. The initiative demonstrated the potential of health professional‐led, crowdfunded activities to enhance public health programs and underscored the opportunity for grassroots health professionals to undertake activities to address existing barriers and ensure broad access and effective delivery of these essential services.

This CSA development is not without limitations. It required time investment, including both paid and volunteer work hours, which may limit its scalability or replication in other settings without similar levels of commitment. Broadly, the CSA alone may not have been sufficient to influence later stages of behavioural change, such as sustained health‐care‐seeking behaviours. Long‐term behavioural change typically requires a broader, multifaceted approach that combines policy changes, community‐based initiatives and integration with health services.^8^ Finally, while the campaign provided estimates of potential reach through social media analytics and media coverage, further evaluation is required to measure actual changes in public awareness and any observable impact on service uptake. Without adequate resources, evaluating the long‐term effectiveness of the campaign may be challenging.

## 4 CONCLUSIONS

This CSA campaign illustrated the potential of health professional initiatives to raise awareness of government health programs. By leveraging crowdfunding and community support, this activity demonstrated a model for other health professionals seeking to promote similar health promotion and awareness initiatives.

## FUNDING INFORMATION

The authors gratefully acknowledge the contributions of the 250 individuals (pharmacists and community members) who contributed to the GoFundMe page. The Media Collective provided their time to undertake this activity at cost price, and the pharmacists, general practitioners and patients donated their time to contribute to the filming.

## CONFLICT OF INTEREST STATEMENT

DH, EH, AT, BH, KH, PR, AB, TM, FY, BC, KP, RT, DL, MA and AP are credentialed pharmacists who are practising pharmacists undertaking home medicines reviews. AP is an Associate Editor of the Australasian Journal on Ageing.

## Data Availability

Raw data are available to all members of the *Consultant Pharmacists Australia* community of practice. Further data that support this study may be available upon reasonable request from the corresponding author.

## References

[1] Lim R , Ellett LMK , Semple S , Roughead EE . The extent of medication‐related hospital admissions in Australia: a review from 1988 to 2021. Drug Saf. 2022;45(3):249‐257. 35089582 10.1007/s40264-021-01144-1 PMC8933367

[2] Lee K , Kouladjian O'Donnell L , Cross AJ , Hawthorne D , Page AT . Clinical pharmacists' reported approaches and processes for undertaking home medicines review services: a national survey. Arch Gerontol Geriatr. 2023;109:104965. doi:10.1016/j.archger.2023.104965 36821873

[3] Weir KR , Naganathan V , Bonner C , et al. Pharmacists' and older adults' perspectives on the benefits and barriers of home medicines reviews – a qualitative study. J Health Serv Res Policy. 2019;25(2):77‐85. doi:10.1177/1355819619858632 31505975

[4] Dhillon AK , Hattingh HL , Stafford A , Hoti K . General practitioners' perceptions on home medicines reviews: a qualitative analysis. BMC Fam Pract. 2015;16(1):16. doi:10.1186/s12875-015-0227-8 25881287 PMC4332443

[5] Services Australia . Requested Medicare items processed from July 2023 to June 2024. Accessed July 25, 2024. http://medicarestatistics.humanservices.gov.au/statistics

[6] Department of Health and Aged Care . Community pharmacy agreements (CPAs) – program data – agreements 4 to 7 2024. Accessed July 10, 2024. https://www.health.gov.au/resources/publications/community‐pharmacy‐agreements‐cpas‐program‐data‐agreements‐4‐to‐7

[7] White L , Klinner C , Carter S . Consumer perspectives of the Australian home medicines review program: benefits and barriers. Res Soc Adm Pharm. 2012;8(1):4‐16. doi:10.1016/j.sapharm.2010.11.003 21493164

[8] Wakefield MA , Loken B , Hornik RC . Use of mass media campaigns to change health behaviour. Lancet. 2010;376(9748):1261‐1271. doi:10.1016/s0140-6736(10)60809-4 20933263 PMC4248563

[9] Turner JP , Currie J , Trimble J , Tannenbaum C . Strategies to promote public engagement around deprescribing. Ther Adv Drug Saf. 2018;9(11):653‐665. doi:10.1177/2042098618794165 30479740 PMC6243424

[10] Brown DR , Soares J , Epping JM , et al. Stand‐alone mass media campaigns to increase physical activity: a community guide updated review. Am J Prev Med. 2012;43(5):551‐561. doi:10.1016/j.amepre.2012.07.035 23079180

[11] Cavill N , Bauman A . Changing the way people think about health‐enhancing physical activity: do mass media campaigns have a role? J Sports Sci. 2004;22(8):771‐790. doi:10.1080/02640410410001712467 15370487

[12] Ghahramani A , de Courten M , Prokofieva M . The potential of social media in health promotion beyond creating awareness: an integrative review. BMC Public Health. 2022;22(1):2402. doi:10.1186/s12889-022-14885-0 36544121 PMC9770563

[13] Prochaska JO , Velicer WF . The transtheoretical model of health behavior change. Am J Health Promot. 1997;12(1):38‐48. doi:10.4278/0890-1171-12.1.38 10170434

[14] Thrul J , Klein AB , Ramo DE . Smoking cessation intervention on Facebook: which content generates the best engagement? J Med Internet Res. 2015;17(11):e244. doi:10.2196/jmir.4575 26561529 PMC4704894

[15] Department of Finance . Community Service Announcement. 2024. Accessed August 26, 2024. https://structure.gov.au/legislation/broadcasting‐services‐act‐1992/community‐service‐announcement

[16] Swain L , Barclay L . Medication reviews are useful, but the model needs to be changed: perspectives of aboriginal health service health professionals on home medicines reviews. BMC Health Serv Res. 2015;15(1):366. doi:10.1186/s12913-015-1029-3 26357987 PMC4566399

